# Evaluating the Clinical Efficacy of Membrane-Assisted Regenerative Therapy in Peri-Implantitis Management: A Comprehensive Review Incorporating Systematic Review Evidence

**DOI:** 10.3390/ma18225227

**Published:** 2025-11-18

**Authors:** Young Joon Cho, Yong Tak Jeong, Hyun Nyun Woo, Hyun Woo Cho, Min Gu Kang, Sung-Min Hwang, Jae-Mok Lee

**Affiliations:** 1 Mac Dental Clinic, Daegu 42117, Republic of Korea; macdental@knu.ac.kr (Y.J.C.); makai29@knu.ac.kr (Y.T.J.); 2 Department of Periodontology, School of Dentistry, Kyungpook National University, Daegu 41940, Republic of Korea; 3 Private Practice, New York, NY 10075, USA; hw2719@cumc.columbia.edu; 4 Department of Oral & Maxillofacial Surgery, Boston University Henry M. Goldman School of Dental Medicine, Boston, MA 02118, USA; dancho99@bu.edu; 5 Private Practice, Gumi-si 39236, Republic of Korea; dent0000@hanmail.net

**Keywords:** peri-implantitis, decontamination, guided bone regeneration, resorbable membranes, non-resorbable membranes, bone regeneration, re-osseointegration

## Abstract

Peri-implantitis (PI) is characterized by inflammatory tissue destruction and alveolar bone loss surrounding dental implants, posing clinical challenges. To promote bone regeneration, clinicians often use resorbable or non-resorbable membranes in combination with bone grafts or biologic agents. Despite their widespread application in PI management, the clinical efficacy of these approaches remains uncertain. Therefore, this study aims to evaluate the role of membrane-assisted regenerative therapy in the management of PI. A systematic literature search was conducted in PubMed, Scopus, Cochrane Library, and Google Scholar following the Preferred Reporting Items for Systematic Reviews and Meta-Analyses (PRISMA 2020) guidelines, with the protocol registered in PROSPERO (CRD420251089276). Sixty-nine studies met the inclusion criteria. The primary outcomes assessed were bone-fill gain and reduction in probing pocket depth (PPD). Although some studies reported improved bone-fill and PPD reduction with membrane-assisted regenerative therapy, the findings were not consistently significant. Future research should validate the clinical efficacy of membranes through well-designed randomized trials and develop advanced decontamination techniques and implant surface modifications that could enhance treatment predictability and patient outcomes. Overall, while membranes show potential clinical value in regenerative therapy, their necessity remains uncertain owing to variability in the current evidence.

## 1. Introduction

Peri-implantitis (PI), a pathological condition caused by the accumulation of dental plaque around dental implants, results in inflammation of the peri-implant mucosa and progressive deterioration of the supporting bone [1]. The accumulation of bacterial biofilm and the corresponding host immune response are the primary etiologic factors, placing patients with poor plaque control and infrequent maintenance visits at higher risk. Anti-infective interventions can reduce soft tissue inflammation and slow the progression of PI. However, once PI develops, complete decontamination of the infected implant surface is challenging, and the restoration of lost bone and re-osseointegration remains a clinical challenge. Despite advances in scaffold-based approaches for regenerating periodontal and peri-implant tissues [2], a standardized and consistently effective treatment protocol is still lacking. Similarly to periodontitis treatment, PI management includes nonsurgical and surgical therapies, with the latter further categorized into resective and reconstructive procedures. Among these, reconstructive therapy aligns with the primary objective of PI management—regeneration of the supporting peri-implant tissues. Traditional regenerative techniques, including guided bone regeneration (GBR), remain the primary approach [3,4]. However, studies investigating the role of membranes as essential components of GBR in PI remain limited, with no consensus reached regarding their optimal application. Previous research has primarily focused on membrane type, with limited consideration of other clinical factors influencing regenerative outcomes [3,4]. In contrast, the present review incorporates additional variables, including keratinized mucosa (KM) width, surface decontamination protocols, and prosthesis management during surgery. Therefore, this review aims to comprehensively assess the clinical effectiveness of membrane-assisted regenerative therapy in PI management. It examines recent advancements in surgical procedures and biomaterials, evaluating their efficacy in preclinical and clinical studies and highlighting the advantages and limitations of various graft materials and membrane-assisted approaches.

Despite the increasing number of clinical studies, evidence-based guidance remains limited regarding how membrane type, peri-implant soft tissue conditions, and decontamination techniques collectively influence clinical outcomes. Furthermore, previous reviews have primarily focused on membrane type alone, without integrating these modifying variables. Accordingly, a more comprehensive analysis that reflects the complexity of clinical decision-making in peri-implant regenerative surgery is needed [5]. Therefore, this review aims to address this gap by systematically integrating clinical and biological variables, including membrane type, defect morphology, KM width, and surface decontamination methods, into a unified framework for evaluating peri-implant regenerative outcomes.

## 2. Materials and Methods

### 2.1. Study Design

This comprehensive review aims to examine the effect of membrane-assisted regenerative therapy on clinical outcomes in the management of PI.

### 2.2. Protocol and Registration

**Figure 1:**
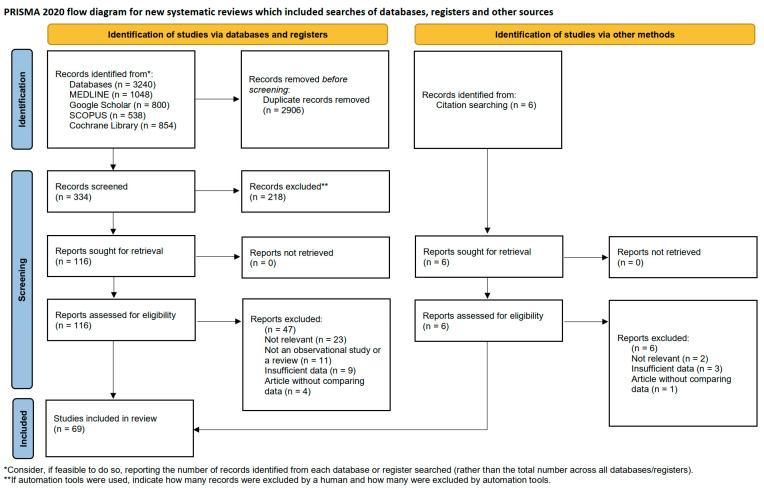
PRISMA flow diagram illustrating the identification, screening, eligibility assessment, and inclusion of studies in this comprehensive review.

This study was conducted following the Preferred Reporting Items for Systematic Reviews and Meta-Analyses (PRISMA) guidelines to ensure transparency in study selection and reporting (Figure 1). Table S1 provides the completed PRISMA checklist, which was used to ensure compliance with established reporting standards [6]. This study relied exclusively on previously published data; therefore, ethical approval and informed patient consent were not required. The study was registered with the International Prospective Register of Systematic Reviews (PROSPERO; registration ID: CRD420251089276). No additional formal protocol was prepared beyond the PROSPERO registration. A minor amendment was made after registration to expand the inclusion criteria, allowing the addition of preclinical (in vivo animal) studies that directly evaluated membrane-assisted regenerative therapy. All other components of the protocol remained unchanged. The PROSPERO registration entry outlines the study objective, inclusion and exclusion criteria, and planned outcomes.

### 2.3. PICO

#### 2.3.1. Population

Patients diagnosed with PI.

#### 2.3.2. Intervention

Membrane-assisted regenerative therapy for management of PI.

#### 2.3.3. Comparison

Comparison between membrane-assisted and non–membrane-assisted regenerative therapy.

#### 2.3.4. Outcome

The primary outcomes were radiographic bone-fill gain and PPD reduction, representing key indicators of peri-implant tissue healing.

The secondary outcomes included changes in KM width, prosthesis management, and the adjunctive application of implantoplasty as modifiers of regenerative outcomes.

### 2.4. Search Strategy

Table S2 provides the details of the electronic search conducted across four databases—PubMed, Scopus, Google Scholar, and the Cochrane Library—on 21 January 2025 MeSH terms and related keywords associated with peri-implantitis, regenerative, reconstructive, and membrane were used during search process. The inclusion period for the data spanned from 1990 to 2024, and the language was limited to English. The search strategy was as follows:

#### 2.4.1. Medline via PubMed

The search terms used were: (periimplantitis OR peri-implantitis OR peri-implant infection OR peri-implant disease OR peri-implant defect OR peri-implant inflammation) AND (regenerative OR reconstructive) AND (surgery OR surgical OR membrane OR collagen membrane OR PTFE) NOT (Review) (Filter: English).

#### 2.4.2. Cochrane Library

The search terms used were: (periimplantitis OR peri-implantitis OR peri-implant infection OR peri-implant disease OR peri-implant defect OR peri-implant inflammation) AND (regenerative OR reconstructive) AND (surgery OR surgical OR membrane OR collagen membrane OR PTFE) NOT (Review).

#### 2.4.3. Scopus

The search terms used were: (TITLE-ABS-KEY (periimplantitis OR peri-implantitis OR peri-implant infection OR peri-implant disease OR peri-implant defect OR peri-implant inflammation) AND TITLE-ABS-KEY (regenerative OR reconstructive) AND TITLE-ABS-KEY (surgery OR surgical OR membrane OR collagen membrane OR PTFE) AND NOT TITLE-ABS-KEY (Review)).

#### 2.4.4. Google Scholar

The search terms used were: (“periimplantitis” OR “peri-implantitis” OR “peri-implant infection” OR “peri-implant disease” OR “peri-implant defect” OR “peri-implant inflammation”) AND (“regenerative” OR “reconstructive”) AND (“surgery” OR “surgical” OR “membrane” OR “collagen membrane” OR “PTFE”).

The search results were subsequently filtered to include “Review articles” using the built-in filtering function of the database. Articles relevant to the study purpose not retrieved through the electronic search were also identified through a manual search conducted by two reviewers (Y.J.C. and Y.T.J.).

### 2.5. Study Selection, Assessment and Agreement

A total of 3240 records were retrieved from electronic databases, including MEDLINE (*n* = 1048), Google Scholar (*n* = 800), Scopus (*n* = 538), and the Cochrane Library (*n* = 854). After removing 2906 duplicate records, 334 unique records were screened based on their titles and abstracts. Of these, 218 records were excluded for being irrelevant to the study objectives.

A total of 116 full-text reports were assessed for eligibility. Of these, 47 were excluded for the following reasons: not being observational studies or reviews (*n* = 11), insufficient data (*n* = 9), lack of comparative data (*n* = 4), or other irrelevance (*n* = 23). Six additional records identified through citation searching were also screened but excluded for similar reasons: not relevant (*n* = 2), insufficient data (*n* = 3), and lack of comparative data (*n* = 1).

Ultimately, 69 studies met the eligibility criteria and were included in the final review. Figure 1 illustrates the complete selection process.

Screening was performed independently by two reviewers (Y.J.C. and Y.T.J.), starting with titles and abstracts, followed by full-text assessment. Discrepancies were resolved through discussion until consensus was achieved; studies were excluded if consensus could not be reached.

#### 2.5.1. Inclusion Criteria

Table S2 provides the list of studies included in this review. These studies were selected based on predefined eligibility criteria applied to all retrieved records. Eligible studies included prospective or retrospective follow-up studies, clinical trials, cohort studies, case–control studies, and case series. Studies were considered if they provided a clear definition of PI and directly compared regenerative surgical outcomes with or without the use of membranes. In the absence of previous examination data, PI was defined as the presence of bleeding and/or suppuration upon gentle probing, PPD ≥ 6 mm, and bone levels ≥ 3 mm apical to the most coronal portion of the intraosseous part of the implant [7].

#### 2.5.2. Exclusion Criteria

The exclusion criteria included studies published in languages other than English, studies with inaccessible data (inability to contact the authors), studies conducted on implant surfaces other than titanium, studies providing insufficient or non-comparative data, and those lacking a clear definition of PI.

Owing to the heterogeneity of study designs, defect characteristics, and outcome measures, this review was conducted using a comprehensive descriptive approach. For each clinical factor of interest (membrane-assisted regenerative therapy, KM width, decontamination methods, prosthesis removal), relevant outcomes were summarized descriptively (Table S2). No formal meta-analysis or predefined subgroup analyses were performed; thus, comparative interpretations should be considered exploratory and interpreted with caution.

### 2.6. Risk-of-Bias Assessment

The Cochrane Risk-of-Bias (RoB) assessment method was used to evaluate the risk of bias in the included studies [8], which evaluates domains such as randomization, blinding, and incomplete outcome data. Six specific domains were evaluated: random sequence generation, allocation concealment, blinding of participants and personnel, blinding of outcome assessment, incomplete outcome data, and selective reporting. Each domain was assessed for risk of bias and classified as low, unclear, or high. Two reviewers (Y.J.C. and Y.T.J.) independently evaluated all included studies, and discrepancies were resolved through discussion. Figure S1 presents a summary of the risk of bias assessments.

#### Risk-of-Bias Evaluation in Included Studies

A risk of bias assessment was performed for the 18 randomized controlled trials (RCTs) included in this review. Figure S1 illustrates that most trials exhibited a low risk of bias in random sequence generation, detection bias (blinding of outcome assessment), attrition bias (incomplete outcome data), and selective reporting. These findings indicate that the methodological rigor regarding randomization and data management was generally adequate across the included studies.

However, allocation concealment and blinding of participants and personnel were frequently rated as high or unclear risk of bias, which reflects the inherent limitations of surgical interventions where complete blinding is rarely feasible. While this bias is difficult to eliminate in clinical surgical studies, it may still influence subjective outcome measures, including PPD reduction and radiographic bone-fill.

Overall, the methodological quality of the included RCTs was acceptable, with the majority demonstrating robust study design and consistent reporting. Given the high or unclear risk associated with allocation and performance bias, the pooled evidence should be interpreted with caution, highlighting the need for more rigorously blinded and transparently reported trials in future research. In interpreting the clinical outcomes within the Section 5 and Section 10, these bias profiles were carefully considered to ensure that the strength of each finding reflected the quality of the underlying study.

### 2.7. Data Analysis

A descriptive comparative analysis was performed using studies that reported bone-fill gain and PPD reduction in comparable quantitative formats (mean ± standard deviation (SD) or convertible pre- and post-treatment values).

For each group (membrane-assisted vs. non-membrane-assisted regenerative therapy), the mean values, standard deviations, and 95% confidence intervals (CIs) were calculated when available. Owing to substantial heterogeneity in study designs and outcome measures, a formal meta-analysis was not conducted.

However, an exploratory pooled quantitative analysis was performed using RevMan version 5.4 (The Cochrane Collaboration, London, UK) to visualize overall trends in bone-fill gain and PPD reduction between treatment modalities. Weighted mean differences (WMDs) and 95% CIs were calculated descriptively under a random-effects model for visualization purposes only, and heterogeneity was expressed as I^2^, with values greater than 75% considered substantial. The pooled results and forest plots were interpreted cautiously due to methodological variability across studies.

Additionally, to assess the efficacy of implantoplasty, a focused subgroup analysis was conducted in clinical studies with a 12-month follow-up that employed membrane-assisted regenerative therapy using resorbable membranes. For this subgroup, Welch’s *t*-test (GraphPad Prism, version 10.0 (GraphPad Software, San Diego, CA, USA)) was used to evaluate the between-group differences (implantoplasty vs. no implantoplasty) in bone-fill gain and PPD reduction.

## 3. Pathophysiology of Peri-Implantitis

**Figure 2:**
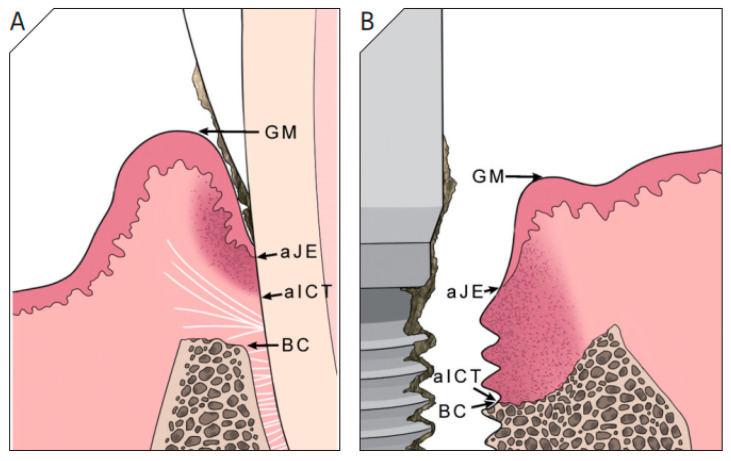
Differences in the short-term progression of inflammatory infiltration into connective tissue between experimental periodontitis (**A**) and PI (**B**) in dogs. In a natural tooth, the supracrestal fiber separates the connective tissue from the alveolar bone, while this structure is absent around a dental implant, allowing inflammation to directly involve the alveolar bone and extend into the bone marrow (adapted from Lindhe et al., 1992 [16]). Abbreviations: GM, Gingival Margin; aJE, Apical extent of Junctional Epithelium; aICT, Apical extent of Inflammatory Cell Tissue; BC, Bone Crest.

PI primarily arises from a bacterial biofilm-induced inflammatory response, resulting in inflammation and bone loss around dental implants [7]. Clinically, PI is characterized by progressive bone loss, bleeding on probing (BOP), and/or suppuration [9]. Inadequate plaque control and local prosthetic designs that impede effective oral hygiene promote infiltration of inflammatory immune cells in peri-implant soft tissues [10]. Continuous plaque control remains crucial during progression of PI, similar to its role in periodontitis [11,12,13]. Additional factors contributing to PI include excessive occlusal overload and the release of titanium particles from the implant surface [14,15]. In contrast to natural teeth, dental implants lack a perpendicular connective tissue fiber attachment, predisposing them to extensive inflammatory infiltration and accelerated bone loss [16,17,18,19] (Figure 2). Additionally, PI lesions are larger and exhibit higher densities of immune cells [20,21]. While PI shares several features with periodontitis, it also displays distinct immunopathological mechanisms, highlighting the need for further research into effective regenerative strategies.

## 4. Regenerative Therapeutic Approaches for Peri-Implantitis

Nonsurgical management of peri-implant bone defects often produces unpredictable outcomes owing to limited access to the implant surface, necessitating surgical intervention [22]. The surgical strategies for addressing peri-implant bone defects include resective, regenerative, and combined methods [23]. Treatment selection should be based on soft tissue conditions, defect morphology, and esthetic considerations [24]. Regenerative surgical procedures generally proceed as follows. First, a flap is raised to expose the contaminated implant surface, typically via a horizontal sulcular incision around the implant prosthesis or healing abutment, extending mesiodistally, with vertical incisions added as necessary [25,26]. A full-thickness flap is elevated, generally limited to either the buccal or lingual side. Inflammatory granulation tissue is carefully removed using curettes or rotary instruments. To facilitate bone regeneration, the exposed defect surface is perforated with a small round bur. The contaminated implant surface is subsequently decontaminated using various methods, and the defect is filled with bone graft material. In cases where the prosthesis is retained, the membrane is adapted around the implant neck [27]. For submerged cases, a cover screw is placed, and the membrane extends 2–3 mm beyond the defect margins [28]. Suturing should ensure proper flap adaptation around gingival penetrations, including prostheses or healing abutments, or achieve primary closure if the prosthesis is removed. Periosteal-releasing incisions may be performed as required.

## 5. Effects of Membrane-Assisted Regenerative Therapy

Bone regeneration and re-osseointegration in PI defects remain clinical challenges [29]. Recent studies have demonstrated the efficacy of treatment strategies adapted from guided tissue regeneration techniques used in periodontal therapy, involving various membrane types, bone grafts, and biologic materials for PI defects [25]. However, the clinical efficacy of membrane-assisted regenerative therapy in PI management remains uncertain [7,12].

Two primary membrane types are used—resorbable and non-resorbable membranes—often with or without bone grafts. Resorbable membranes—including collagen-based membranes (Bio-Gide^®^ (Geistlich Pharma AG, Wolhusen, Switzerland)) [25,30,31,32,33,34,35,36,37,38], synthetic polymers (OsseoGuard^®^ (Zimmer Biomet, Warsaw, IN, USA)) [4,39], and concentrated growth factor (CGF) matrices [32]—undergo gradual degradation and are resorbed by the body [40]. Their primary advantages include the elimination of a second surgical procedure for membrane removal, reduced postoperative complications, and improved patient comfort. A major limitation of resorbable membranes is susceptibility to premature exposure and degradation, which may compromise barrier function [40]. In contrast, non-resorbable membranes, including expanded polytetrafluoroethylene (e-PTFE) [25,27] membranes (Gore-Tex, W. L. Gore & Associates, Newark, DE, USA), provide greater long-term stability and structural integrity, thereby enhancing their ability to contain and support tissue regeneration, particularly in complex bony defects [40]. Nevertheless, the requirement for a secondary removal surgery and the increased risk of infection following early exposure remain major drawbacks [40].

Numerous studies have reported the effects of membrane-assisted regenerative therapy, typically used in combination with bone grafts and/or biologics in the surgical management of PI (Table 1). The use of bone grafts alone improves both bone-fill and PPD reduction in several studies, although the reported outcomes vary [41]. Among these, autogenous bone grafts were associated with the highest gains in bone-fill and PPD reduction [25,37,42,43,44]. The addition of biologics to bone grafts without membrane-assisted regenerative therapy further enhanced bone-fill and PPD reduction compared with bone graft alone; however, the available data were limited [45]. The most common approach involves the combination of resorbable membranes with bone grafts [4,25,31,32,33,34,37,38,39,44,46,47,48,49,50,51,52]. While results vary depending on the graft material used, these combinations generally produce outcomes comparable to or less effective than those observed without membrane-assisted regenerative therapy. The addition of biologics to membranes and bone grafts produced moderate improvements in PPD reduction and bone-fill [30,35,36,53,54,55,56]. Additionally, non-resorbable membranes combined with bone grafts demonstrate promising results [57]; however, the available evidence remains limited compared to that of resorbable membranes [25,58,59].

The mean bone-fill gain and PPD reduction across different treatment modalities were compared (Table 2).

**Figure 3:**
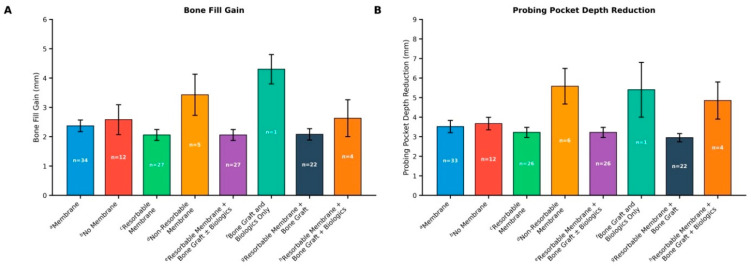
Comparison of Average Bone-Fill Gain and PPD Reduction among Different Treatment Approaches. (**A**). Average Bone Fill Gain. (**B**). PPD Reduction. Error bars represent ± SD, indicating variability across the included studies. ^a^ studies using any membrane type; ^b^ studies without membranes; ^c^ studies with resorbable membranes; ^d^ studies with non-resorbable membranes; ^e^ studies combining resorbable membranes and bone graft with or without biologics; ^f^ studies using bone grafts with or without biologics; ^g^ studies combining resorbable membranes and bone grafts; ^h^ studies combining resorbable membranes, bone graft, and biologics. For the bone graft and biologics-only group, values were extracted from a single study; therefore, CIs represent within-study variance only and should be interpreted with caution. Abbreviations: PPD, probing pocket depth; SD, standard deviation.

Comparisons were made between membrane-assisted and non-membrane-assisted regenerative therapies; between bone-graft- or biologic-only procedures and those incorporating membrane-assisted regenerative therapy; and between resorbable membranes combined with bone grafts, with or without biologic adjuncts. These comparisons indicated similar or greater bone-fill gain outcomes (Figure 3A). Most treatments showed similar PPD reductions, with the combination of resorbable membranes, bone grafts, and biologics producing the greatest improvement (Figure 3B).

Although our analysis revealed membranes use in enhancing bone-fill and PPD reduction, some limitations must be acknowledged. The studies included in this review varied in their study designs, inclusion and exclusion criteria, and outcome measurement standardization, which may limit the generalizability of the findings. Moreover, the lack of meta-analytic data precludes formal statistical comparison between groups. Future studies should use standardized outcome measures and rigorous study designs to clarify the efficacy and indications of membrane-assisted regenerative therapy in PI.

An exploratory pooled analysis was performed using 14 RCTs with 12-month follow-up data selected from the 18 RCTs that met the inclusion criteria. The pooled findings suggested that membrane-assisted regenerative therapy yielded greater bone-fill gain and PPD reduction than non-membrane approaches (Figure S2). However, substantial heterogeneity was observed (I^2^ > 80%), likely due to variations in defect morphology, membrane type, and decontamination methods. Publication bias was evaluated using funnel plots (Figure S3). Panels A and B illustrate the distribution of studies for bone-fill gain and PPD reduction, respectively. Data points representing membrane-assisted (regenerative therapy) and non-membrane (bone-graft-only) groups are symmetrically scattered around the mean difference axis, and the inverted funnel shape appears balanced, indicating minimal publication bias. Because the analysis was exploratory rather than confirmatory, these pooled estimates should be interpreted as visual trends rather than statistically validated meta-analytic outcomes. Further randomized trials with standardized methodology are needed to confirm these observations.

## 6. Role of Keratinized Mucosa in Membrane-Assisted Regenerative Therapy

PI, a major complication of dental implants, is characterized by inflammation of the surrounding tissues that causes progressive bone loss and may lead to implant failure [7,70]. The presence of KM is essential for the biological stability and clinical management of PI. Several studies have shown that KM is critical for improving treatment outcomes [70,71,72]. In this section, the effects of KM on bone-fill gain and PPD reduction are evaluated in membrane-assisted regenerative therapy for PI (Table 3).

A KM width of ≥2 mm has been associated with greater PPD reduction and enhanced bone-fill in treatments combining membranes and bone grafts [44,47,48,72,73,74]. These improvements are observed across different membrane types and bone graft materials. Although available data are limited, a KM width of <2 mm is associated with less favorable treatment outcomes, including modest bone-fill gains [72]. A KM width of ≥2 mm is thought to promote soft-tissue stability, strengthen the epithelial seal, and maintain a protected environment around the membrane, collectively enhancing bone regeneration. Nevertheless, additional clinical and histological evidence is required to confirm this protective mechanism.

Although our analysis highlights the importance of KM width in membrane-assisted regenerative therapy for PI management, some limitations must be acknowledged. Variations in study design, surgical techniques, and outcome assessment limit the reliability of these findings. Additionally, the lack of meta-analytic methods limits our ability to quantify the statistical significance and consistency of effects across studies. Furthermore, interactions between KM width and clinical variables—including membrane type, surgical approach, and maintenance therapy—remain unclear because of heterogeneous study designs and limited reporting. Therefore, future studies should adopt standardized protocols and quantitative analyses to clarify the role of KM and define clinical thresholds for optimizing regenerative outcomes in PI management.

## 7. Efficient Decontamination Methods in Membrane-Assisted Regenerative Therapy

**Figure 4:**
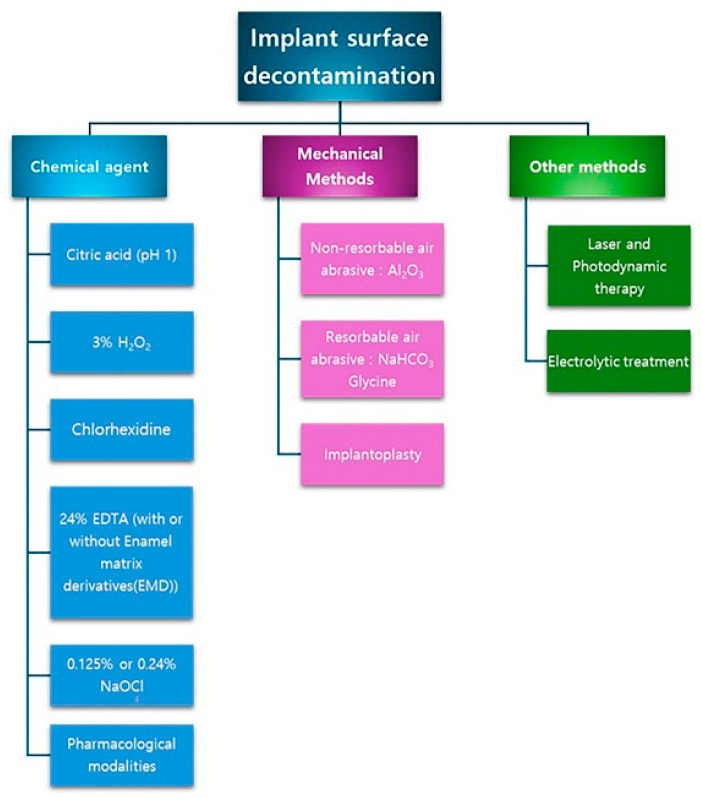
Classification of implant surface decontamination strategies into three categories (mechanical, chemical, and other methods).

Effective decontamination of implant surfaces is essential for achieving successful re-osseointegration. Monje et al. [75] classified implant surface decontamination methods into three main categories—mechanical, chemical, and other—as illustrated in Figure 4. In reconstructive therapy, strict decontamination protocols are required because even minimal surface contamination can impair regeneration.

### 7.1. Mechanical Decontamination Methods

**Figure 5:**
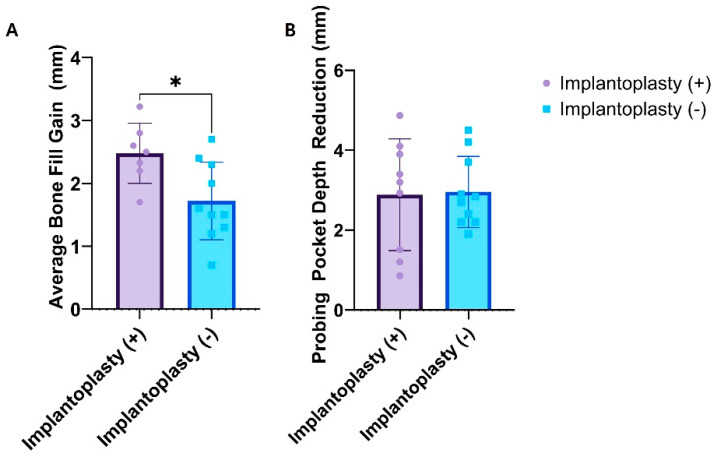
Comparative clinical outcomes between implantoplasty-treated (+) and non-treated (−) groups in membrane-assisted regenerative therapy, based on human clinical studies with 12-month follow-up and use of resorbable membranes. (**A**) Average bone-fill gain and (**B**) PPD reduction. Each dot represents the mean value reported in an individual study; bars indicate the group mean ± standard deviation. The implantoplasty (+) group demonstrated a significantly higher average bone-fill gain (* *p* < 0.05, Welch’s *t*-test), while the difference in PPD reduction between groups was not statistically significant. Abbreviation: PPD, probing pocket depth.

- Air-Abrasive Techniques: The traditional use of Al_2_O_3_ particles has raised concerns about their potential embedding in surrounding tissues. Absorbable powders, such as glycine, NaHCO_3_, and CaCO_3_, are now preferred alternatives. Among them, glycine shows high efficacy with minimal implant surface damage. However, complete decontamination remains difficult, particularly in implant thread valleys.
- Implantoplasty: This method mechanically smooths contaminated implant threads to reduce plaque accumulation and surface roughness. A 9-year follow-up study reports an 89% success rate [76]. However, potential limitations include heat generation, structural weakening of the implant, and titanium particle release, which may contribute to peri-implant inflammation. Despite the lack of a clear consensus and ongoing debate over its efficacy, implantoplasty is regarded as a beneficial adjunctive mechanical decontamination procedure that promotes bone-fill and reduces PPD in PI management. Regular post-treatment maintenance is essential for sustaining long-term outcomes (Table 4 & Figure 5).
- The subgroup analysis of 12-month clinical studies using resorbable membranes revealed a quantitative trend favoring implantoplasty (Figure 5). The implantoplasty (+) group showed a significantly greater mean bone-fill gain than the implantoplasty (–) group (*p* < 0.05, Welch’s *t*-test), whereas the PPD reduction difference was not statistically significant. This finding suggests that implantoplasty mainly promotes hard-tissue regeneration by creating a smoother and cleaner implant surface that facilitates clot stabilization and bone matrix deposition beneath the membrane. In contrast, soft tissue changes such as pocket reduction appear more influenced by keratinized mucosa width, surgical access, and maintenance than by surface modification alone. In this standardized 12-month resorbable-membrane cohort, implantoplasty appeared to be an effective mechanical decontamination adjunct that enhances the predictability of guided bone regeneration. Nevertheless, some limitations should be acknowledged. The included studies varied in design, defect morphology, adjunctive biologics, decontamination methods, and maintenance protocols, resulting in inevitable heterogeneity. In addition, this analysis was based on study-level mean values rather than patient-level data; therefore, its findings should be interpreted cautiously as hypothesis-generating rather than definitive meta-analytic conclusions. These bone-fill trends are hypothesis-generating and require prospective, controlled trials with standardized protocol.

### 7.2. Chemical Decontamination Methods

Chemical agents are commonly used for enhancing the effectiveness of mechanical decontamination methods:

- Citric Acid: Exhibits strong bactericidal activity but may adversely affect tissue regeneration due to its low pH and potential cytotoxicity.
- 3% Hydrogen Peroxide: Reduces inflammation and supports re-osseointegration, particularly when combined with laser therapy.
- Chlorhexidine (CHX): Provides long-term antimicrobial effects but demonstrates limited effectiveness when used alone.
- EDTA (24%): Facilitates the removal of bacterial endotoxins and promotes tissue healing; however, it requires thorough rinsing to mitigate cytotoxic effects.
- Sodium Hypochlorite (NaOCl): Effectively disrupts microbial biofilms; however, its optimal concentration for clinical safety remains under investigation.
- Pharmacological Approaches: Effective bacterial control is critical for managing PI, with antibiotics commonly used as adjuncts to mechanical and chemical decontamination. Adjunctive systemic antibiotics (e.g., amoxicillin + metronidazole) have been reported to improve outcomes in some study [76]. However, antibiotic therapy alone is inadequate when plaque control is poor, as bacterial recolonization can lead to reinfection. Therefore, consistent, supportive PI maintenance is necessary to sustain therapeutic outcomes and prevent recurrence.

### 7.3. Laser-Based Applications

Laser-based therapies (e.g., CO_2_, Er:YAG) can reduce bacterial load with limited surface alteration and may enhance regenerative outcomes; however, long-term clinical evidence is limited, so they are best used as adjuncts. Despite these promising outcomes, long-term clinical evidence remains limited, and laser therapy is currently recommended as an adjunct rather than a standalone treatment. Photodynamic therapy, combining laser light with photosensitizing agents, shows potential as an adjunctive option in PI management.

**Figure 6:**
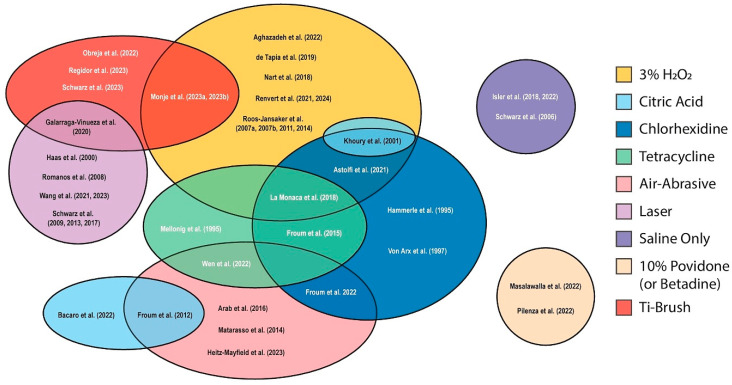
Venn diagram illustrating the overlap among mechanical, chemical, and laser-assisted decontamination methods used in PI management involving membrane-assisted regenerative therapy. Abbreviations: H_2_O_2_, Hydrogen Peroxide; Ti-brush, Titanium Brush [3,4,25,26,27,30,31,32,33,34,35,36,37,38,40,44,46,48,49,50,52,53,56,57,58,59,61,62,63,64,67,69,73,74,77,78,79,81,82,83,84].

Optimal implant surface decontamination requires an integrated strategy incorporating mechanical, chemical, and laser-based methods (Figure 6). Each approach offers distinct strengths and limitations, highlighting the importance of personalized treatment planning.

### 7.4. Comparative Analysis of Decontamination Modalities

No single “gold standard” currently exists for implant surface decontamination. A comprehensive systematic review evaluating mechanical, chemical, and laser-based protocols reports that no single method demonstrates clear evidence of superiority. This finding highlights the need for a more targeted comparison of each specific limitation of the modality. Mechanical debridement remains the essential baseline therapy, but is often insufficient for thoroughly cleaning complex implant topographies. Clinicians often incorporate chemical agents as adjuncts to mechanical debridement to enhance decontamination. However, this practice lacks strong evidence. A recent meta-analysis reported that chemicals, such as CHX, offer no significant clinical benefit over mechanical debridement alone [85]. In contrast, adjunctive laser therapy remains a more dynamic and debated field. Lasers are investigated for their ability to effectively disrupt biofilms. A systematic review supports their adjunctive use [86], reporting greater improvements in key clinical outcomes (PPD reduction, BOP) than mechanical therapy alone. However, evidence supporting the use of lasers remains inconsistent. A separate meta-analysis provides a contrasting perspective [87], reporting that laser-assisted decontamination achieves outcomes similar to those of conventional mechanical therapy, with no statistically significant clinical advantage. This discrepancy in recent studies (Akerzoul et al., 2025 vs. Pisano et al., 2021) supports the previous findings by Baima et al. (2022) [86,87,88]. Currently, no single decontamination modality demonstrates definitive superiority for implant surface treatment. This persistent lack of consensus highlights the critical need for robust, evidence-based research. Standardized, reproducible protocols—as optimized monotherapies or rational multimodal combinations—are needed.

### 7.5. Clinical Implications of Surface Treatments in Membrane-Assisted Regenerative Therapy

The effectiveness of membrane-assisted regenerative therapy depends on the quality of implant surface decontamination. A clean, oxide-intact, and moderately rough surface promotes fibrin adhesion, osteoblast migration, and early extracellular matrix deposition, thereby establishing a stable foundation for bone regeneration under the membrane. Conversely, excessive polishing, uncontrolled acid etching, or thermal damage can disrupt the titanium oxide layer, diminish protein adsorption, and impair osteoconduction, ultimately reducing the predictability of membrane-assisted regenerative therapy. Therefore, decontamination strategies should balance thorough bacterial removal with preservation of the physicochemical surface characteristics supporting cellular adhesion and bone formation. Maintaining this balance is essential for achieving consistent, biologically favorable outcomes in membrane-assisted regenerative therapy.

## 8. Effect of Prosthesis Retention Versus Removal on Membrane-Assisted Regenerative Therapy

The effect of prosthesis removal during membrane-assisted regenerative therapy for PI bone defects remains inadequately investigated. In this section, studies comparing clinical outcomes between submerged and non-submerged healing post-regenerative management for PI are reviewed. Wen et al. reported that submerged healing results in superior outcomes, showing 0.9 mm greater clinical defect fill, 1.7 mm greater radiographic bone-fill, and 1.3 mm greater PPD reduction than the non-submerged healing [68,69,80]. In contrast, in a study involving 32 implants across 28 patients, Astolfi et al. report no significant difference in bone regeneration outcomes between cases with prosthesis removal and those where the prosthesis was retained post-surgery [81]. Similarly, Daugela et al., analyzing 18 systematic reviews, report no significant differences in radiographic bone level changes or PPD reduction between submerged and non-submerged healing sites [89].

Overall, the reported bone-fill values ranged from approximately 1.6 mm to 3.5 mm, while PPD reduction ranged from 1.5 mm to 2.9 mm, regardless of whether the prosthesis was removed (Table 5). Biologically, removing the prosthesis during regenerative therapy potentially facilitates the creation of a sealed and stable healing environment, reducing micromotion and minimizing bacterial infiltration under the membrane. This stability may enhance blood clot maturation and promote optimal adaptation of the barrier membrane, potentially improving early regenerative outcomes. Conversely, maintaining the prosthesis may preserve occlusal function, prosthetic stability, and patient comfort, potentially supporting long-term success when infection control is adequate.

Overall, current evidence suggests that, while prosthesis removal may enhance early tissue stability and membrane adaptation, overall clinical success in membrane-assisted regenerative therapy depends primarily on the effectiveness of surface decontamination, membrane stability, and infection control rather than on prosthesis removal alone. Future studies should further clarify the interaction among prosthesis management, defect morphology, and decontamination protocols to develop standardized clinical guidelines for optimizing regenerative outcomes in PI management.

**Table 5:** Studies Evaluating the Effect of Prosthesis Removal on Clinical Outcomes During Membrane-Assisted Regenerative Therapy for PI management.

Author (Year)	Wen (2022a, 2022b, 2024) [68,69,80]		Astolfi (2021) [81]		Daugela (2016) [89] *	
**Study Model**	Prospective study		Retrospective study		Meta-analysis of systematic literature review	
**Crown Removal**	Removed & Submerged	Removed, healing abutment maintained	Removed & submerged	Crown maintained	Removed & submerged	Crown or healing abutment maintained
**Sample Size** **(patients)**	30 implants (22 patients)	29 implants (24 patients)	32 implants (28 patients)		Deppe et al. (2007) [60] Roos-Jansåker et al. (2007a, 2007b, 2014) [46,63,64] Schwarz et al. (2009, 2010, 2013) [77,82,90] Romanos et al. (2008) [83] Roccuzzo et al. (2011) [91] Froum et al. (2012, 2015) [30,53] Aghazadeh et al. (2012) [92] Wohlfahrt et al. (2012) [66] Wiltfang et al. (2012) [65] Matarasso et al. (2014) [31] Jepsen et al. (2015) [11]	
**Mechanical Debridement**	curettage, implantoplasty, air-powder		curettage, implantoplasty			
**Surface Decontamination**	2.5 mL of 250 mg TC (5 min)		3–5% H_2_O_2_ (2 min)			
**Bone Graft Type**	60% FDBA (Maxgraft^®^), 20% mineralized bovine bone (Cerabone^®^), 20% autobone		Bio-Oss^®^			
**Membrane Type**	dPTFE (Cytoplast^®^)	Collagen membrane (Jason^®^)	collagen membrane (Jason^®^)			
**Follow-up or Re-entry**	8 mo	8–12 mo	2 yr			
**Radiographic Bone-Fill** **(mm)**	3.47 ± 0.41	1.63 ± 1.7	2.18 ± 1.41	2.84 ± 1.78	2.17 (95% CIs 1.87–2.47)	1.91 (95% CIs 1.44–2.39)
**Clinical Bone-Fill** **(mm)**	3.22 ± 0.41	2.33 ± 1.88				
**Probing Pocket Depth Reduction** **(mm)**	2.93 ± 0.25	1.51 ± 1.17			2.68 (95% CIs 1.71–3.64)	2.77 (95% CIs 2.23–3.3)
**BOP Reduction to** **(baseline 100%)**	36.60%	34.50%	41.70%	30%		

* The review included all human prospective and retrospective follow-up studies and clinical trials, cohort studies, case–control studies, and case series studies on surgical regenerative management of PI, published between January 2006 and March 2016. Abbreviations: BOP, bleeding on probing; FDBA, freeze-dried bone allograft; dPTFE, dense polytetrafluoroethylene; mo, month(s); yr, year(s); CIs, confidence intervals. Maxgraft^®^ (botiss biomaterials GmbH, Zossen, Germany), Cerabone^®^ (Botiss Biomaterials GmbH, Zossen, Germany), Cytoplast^®^ (Osteogenics Biomedical, Lubbock, TX, USA), Jason^®^ (Botiss Biomaterials GmbH, Zossen, Germany).

## 9. Histological Insights into Membrane-Assisted and Non-Membrane Regenerative Therapy for Peri-Implantitis Management

Histological evaluation offers valuable insights into the biological response of PI tissues to regenerative interventions. Tissue-level healing outcomes of regenerative treatments were compared in preclinical studies conducted with and without membrane-assisted regenerative therapy (Table 6).

Studies employing non-resorbable membranes, such as e-PTFE, report favorable space maintenance and significant bone-fill. However, these membranes are often attributed to fibrous tissue encapsulation and a higher risk of membrane exposure, both of which may compromise re-osseointegration [93]. In contrast, resorbable membranes demonstrate improved soft-tissue integration and fewer postoperative complications but show greater variability in space maintenance and the predictability of bone regeneration [28,94,95].

In contrast, regenerative treatments performed without membranes often result in limited bone-fill, incomplete defect resolution, and increased epithelial downgrowth and inflammatory cell infiltration [96]. Nevertheless, these outcomes vary significantly and are highly influenced by factors such as defect morphology, the degree of surface decontamination, and the presence of KM.

Collectively, histological findings suggest that the application of barrier membrane facilitates organized bone and connective-tissue regeneration under well-controlled conditions, primarily by maintaining space and preventing soft-tissue invasion. However, these benefits are not consistently observed across all defect types. Ultimately, factors such as surgical access, infection control, and soft-tissue quality exert equal or greater influence on healing outcomes. Therefore, the use of membranes should be customized to defect morphology and clinical context, rather than considered a universally superior strategy for PI regenerative therapy.

**Table 6:** Histologic Studies in Membrane-Assisted Regenerative Therapy.

Animal Type	Membrane Type	Bone Graft Type	Key Histologic Findings	Regenerative Outcomes	Year	Author	Reference
Beagle dog	Non-Resorbable (Gore-Tex^®^)	Resorbable HA	Space maintenance under membrane; organized new bone formation; limited epithelial downgrowth (histologic observations).	Structured bone fills with membrane support.	1997	Hürzeler et al.	[97]
Beagle dog	Resorbable	-	Connective tissue encapsulation around the implant neck; no new bone formation at GTR-treated sites; similar outcome with or without submerging.	No significant bone regeneration or re-osseointegration; GTR ineffective under study conditions	1993	Grunder et al.	[93]
Cynomolgus monkey	Non-Resorbable (Gore-Tex^®^)	Autogenous	New bone formation under the membrane; soft tissue encapsulation when exposed.	Bone-fill present; integration limited when exposure occurs.	2003a	Schou et al.	[94]
Cynomolgus monkey	Non-Resorbable (Gore-Tex^®^)	Autogenous	Mature lamellar bone in protected areas; epithelial migration in unprotected zones.	Stable augmentation with ~45% bone-to-implant contact; partial re-osseointegration.	2003b	Schou et al.	[98]
Cynomolgus monkey	Non-Resorbable (Gore-Tex^®^)	Bio-Oss^®^	Bio-Oss particles integrated within new bone; occlusal particles surrounded by connective tissue; no osteoclastic activity near particles.	Mean bone-to-implant contact 36%; stable bone-fill, slightly less than autogenous bone models.	2003c	Schou et al.	[95]

### Independent Evaluation of Preclinical and Clinical Studies

A stratified analysis of the studies included in this review, classifying them into preclinical and clinical groups, revealed a significant difference in the mean bone-fill gain (Table S3). The weighted mean for the preclinical group was 4.511 mm (95% CIs: 4.433–4.588), significantly higher than the 3.027 mm (95% CIs: 2.650, 3.404) observed in the clinical group. This disparity highlights the variability introduced when combining these two levels of evidence, suggesting that this aggregation potentially limits the direct clinical applicability of the findings.

Preclinical (animal) studies showed the maximum potential efficacy of non-resorbable membranes. However, the pooled SD (0.400) and the narrow 95% CIs for this preclinical group were based on a single study (*n* = 7) that reported SD. This represents a methodological limitation, restricting the ability to reliably assess the consistency of these results.

Theoretically, the high mean value (4.511 mm) observed in animal studies likely reflects the controlled experimental conditions of these models, typically exhibiting uniform biological characteristics and standardized defect morphologies. These findings indicate that, at the tissue level, the biological principles underlying the membrane can function effectively under well-regulated experimental conditions. However, directly extrapolating these ideal outcomes to the complex process of human tissue healing remains challenging.

In clinical (human) studies, a lower mean bone-fill of 3.027 mm was observed, accompanied by a wider, more rigorously calculated CIs and a higher pooled SD (1.666). This finding suggests that, in real-world clinical conditions, several uncontrollable variables influence outcomes, including systemic health, age, smoking status, defect complexity, post-operative care, and individual healing responses of the patient. Thus, while the high success rate observed in animal studies demonstrates the potential for clinical application, a lower mean gain with higher variability represents a more realistic expectation for patient outcomes.

## 10. Conclusions & Future Directions

PI management is currently at a pivotal stage, as emerging evidence increasingly challenges the routine use of membrane-assisted regenerative therapy. Current evidence does not consistently show that membrane-assisted regenerative therapy results in superior clinical outcomes compared to non-membrane-assisted approaches, prompting a re-evaluation of conventional protocols in favor of more targeted, evidence-based treatment strategies. While membranes are widely employed in PI regenerative therapy, their clinical benefits remain limited and inconsistent. Their effectiveness depends on case-specific factors, such as defect morphology and soft-tissue conditions, rather than universal applicability. Moreover, factors such as the width of KM may exert a greater influence on treatment outcomes. Therefore, the use of membranes should be guided by careful, individualized clinical judgment rather than routine application.

### 10.1. Proposed Framework for Clinical Decision-Making in Membrane-Assisted Regenerative Therapy

**Figure 7:**
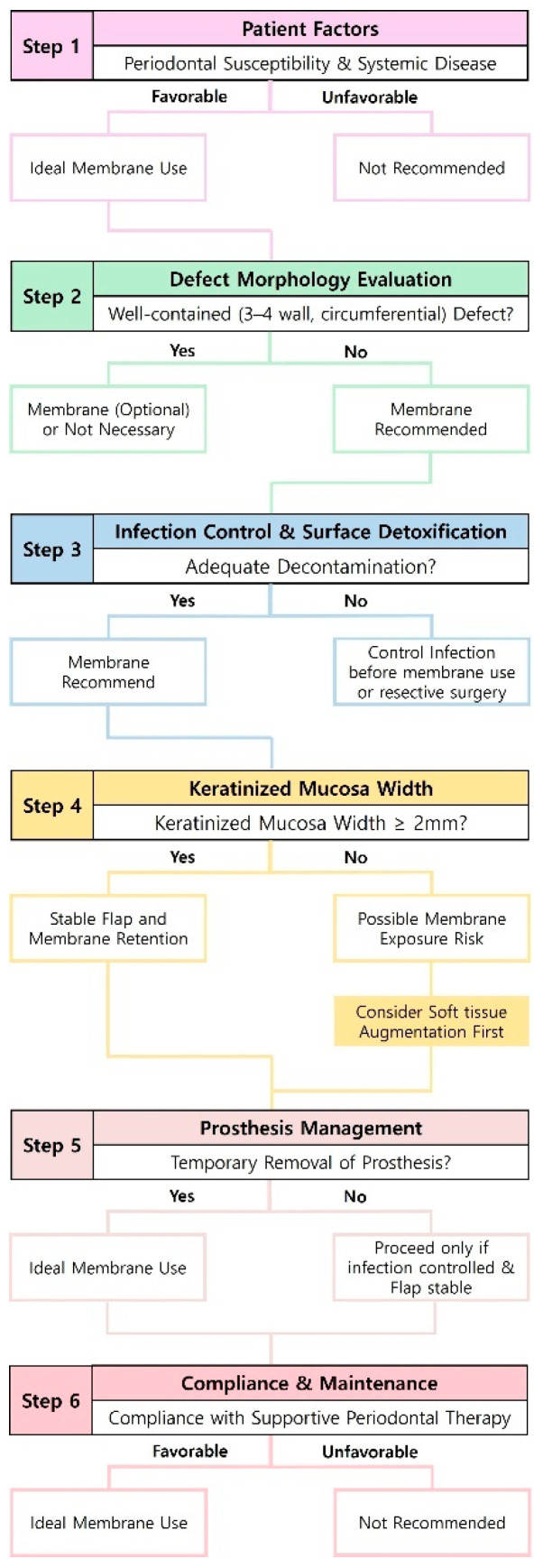
Proposed clinical decision-making algorithm for membrane-assisted regenerative therapy in PI management.

Implementation of membrane-assisted regenerative therapy should follow a case-specific, evidence-based approach rather than a fixed protocol. This review proposes a structured decision-making framework integrating key determinants—defect morphology, KM width, surface decontamination strategy, prosthesis management, and patient-maintenance compliance (Figure 7). The proposed flowchart provides a stepwise guide to determine when membrane-assisted regenerative therapy is indicated or contraindicated in peri-implantitis regenerative surgery, emphasizing the dynamic relationship among tissue conditions, defect configuration, and infection-control parameters.

The diagram summarizes decision pathways based on defect morphology, keratinized-mucosa width, decontamination strategy, prosthesis management, and maintenance compliance.

**Figure 8:**
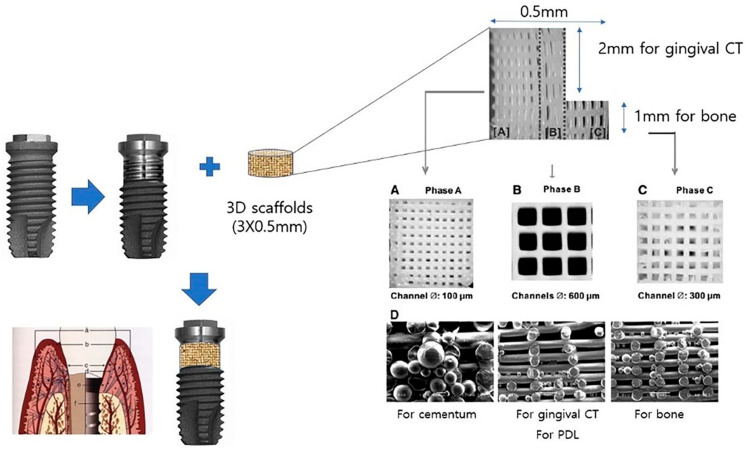
Conceptual schematic of a modular implant fixture designed to replace membranes of uncertain effectiveness in membrane-assisted regenerative therapy for PI management. This proposed design includes a 3D scaffold-based, absorbable, replaceable band located on the upper 3 mm of the implant fixture, a region prone to PI (The 3D scaffold structure was adapted from Cho 2016 [99]). (**A**) Engineering of cementum on the implant surface using 3D-printed polycaprolactone (PCL) scaffolds. (**B**) Engineering of the periodontal ligament on the cementum-coated implant surface using 3D-printed PCL scaffolds. (**C**) Engineering of alveolar bone on the regenerated periodontal ligament using 3D-printed PCL scaffolds. (**D**) 3D-printed PCL scaffolds with embedded growth factors targeting cementum regeneration (left), PCL scaffolds with embedded growth factors targeting periodontal ligament regeneration (center), and PCL scaffolds with embedded growth factors targeting alveolar bone regeneration (right). a: oral epithelium; b: sulcular epithelium; c: junctional epithelium; d: lack of connective tissue attachment; e: hypocellular and hypovascular connective tissue zone adjacent to the implant; f: absence of blood supply to the periodontal ligament. Abbreviations: PDL, Periodontal ligament; CT, Connective tissue; 3D, 3-dimensional.

Furthermore, innovations in implant design, such as modular systems incorporating absorbable three-dimensional (3D) scaffold bands (Figure 8), may represent future alternatives to conventional membranes. These designs could simplify surgical procedures, minimize patient morbidity, and enhance long-term PI stability. Additionally, developing advanced decontamination techniques remains a critical priority, as safe and effective methods are essential for eliminating microbial biofilms from implant surfaces without compromising adjacent tissues.

Advancements in techniques and device innovations could enhance the predictability and long-term durability of PI management while minimizing invasiveness, reducing recovery time, and improving patient-centered outcomes.

### 10.2. Limitations and Future Perspectives

Several limitations must be acknowledged. Because of marked heterogeneity in study designs and reporting, a formal meta-analysis was not feasible, and data synthesis relied primarily on descriptive analysis. Variations in surgical protocols, outcome definitions, and risk-of-bias profiles may further limit the robustness of the conclusions. Although most RCTs exhibited a low risk of bias in domains such as randomization and selective reporting, allocation concealment and blinding of participants or personnel were frequently rated as unclear or high-risk—an inherent limitation in surgical research. Performance bias is particularly relevant for regenerative procedures, in which complete blinding is challenging yet crucial for minimizing subjective interpretation of clinical outcomes such as PPD reduction and radiographic bone-fill. Accordingly, results from low-risk studies were prioritized, whereas evidence from studies with higher bias risk was regarded as exploratory. Overall, the certainty of evidence should be considered moderate, which is consistent with recent experimental and clinical observations reporting the reliability of post-treatment bone-level measurements, the potential benefits of membrane-assisted regenerative therapy, and the influence of biologic and graft-related factors on regenerative outcomes [84,100,101,102,103]. Collectively, these findings underscore the need for future well-designed studies with standardized methodology to clarify the specific indications and long-term predictability of membrane-assisted regenerative therapy. Future clinical studies should adhere to rigorous and transparent methodological standards, incorporating appropriate randomization, allocation concealment, and feasible blinding measures. Such improvements are essential for enhancing reproducibility and clinical applicability of membrane-assisted regenerative therapy in PI management.

In summary, the future of PI management depends on adopting innovative, evidence-based strategies. Advances in implant technology, regenerative biomaterials, and optimized decontamination protocols will allow clinicians to implement more predictable, efficient, and patient-centered approaches for long-term disease control and tissue regeneration.

## Figures and Tables

**Table 1:** Summary of Studies on Bone-Fill Gain and PPD Reduction across Different Membrane Types, Bone Grafts, and Biologic Materials.

Treatment Modality	Membrane Type	Bone Graft Type	Study Model	Sample Size	Average Bone-Fill Gain ± SD (mm)	Average PPD Reduction ± SD (mm)	Period	Year	Author	Reference
**No membrane + Bone Graft Only ± Biologics**	-	Autogenous	Prospective Clinical Study	17 patients with 25 implants	6.2 (SD not available)	3.5 (SD not available)	3 yr	2000	Behneke	[42]
	-	Alloplast (β-TCP)	Prospective Clinical Trial	Nine patients with 17 implants	4.7 ± 1.1	3.8 ± 0.5	5 yr	2007	Deppe	[60]
	-	Autogenous	Clinical Trial	seven patients with 12 implants	3.2 ± 2.4	5.1 ± 2.7	3 yr	2001	Khoury	[25]
	-	Bio-Oss Collagen^®^	Randomized Clinical Trial	22 patients with 22 implants	0.9 ± 1.3	4.2 ± 2.2	1 yr	2023	Regidor	[37]
	-	Xenograft Granules (EndoBon^®^)	Randomized Clinical Trial	21 implants	0.7 ± 0.2	4.0 ± 0.3	1 yr	2018	Renvert	[12]
	-	Bio-Oss Collagen^®^	Case Series	64 patients with 51 implants	Not Available	2.8 ± 0.5	5 yr	2021	Roccuzzo	[43]
	-	Allograft	Randomized Clinical Trial	16 patients with 24 implants	1.7 ± 0.8	4.0 ± 1.5	1 yr	2023a	Monje	[61]
	-	Alloplast (Algipore^®^)	Prospective Case–Control Study	15 patients with 27 implants	1.3 ± 1.3	Not Available	3 yr	2011	Roos-Jansåker	[62]
	-	Alloplast (Algipore^®^)	Prospective Cohort Study	19 patients with 36 implants	1.4 ± 0.4	2.2 ± 0.3	1 yr	2007b	Roos-Jansåker	[63]
	-	Alloplast (Algipore^®^)	Clinical Trial	12 patients with 22 implants	1.1 ± 1.2	3.3 ± 2.1	5 yr	2014	Roos-Jansåker	[64]
	-	Bio-Oss collagen^®^ + Biologics (EMD^®^)	Prospective Cohort Study	30 patients with 30 implants	4.3 ± 0.5	5.4 ± 1.4	3 yr	2018	Mercado	[45]
	-	Autogenous + demineralized xenograft	Prospective Case Series	22 patients with 36 implants	3.5 (95% CIs: 2.7, 4.3) (SD not available)	4.0 (95% CIs: 3.3, 4.6) (SD not available)	1 yr	2012	Wiltfang	[65]
	-	Porous Titanium Granule	Randomized Clinical Trial	16 patients with 16 implants	2.0 ± 1.7	1.7 ± 1.7	1 yr	2012	Wohlfahrt	[66]
**Resorbable membrane + Bone Graft ± Biologics**	Resorbable	Bio-Oss^®^	Clinical Trial	12 patients with 12 implants	1.4 ± 1.1	2.4 ± 1.0	6 mo	2016	Arab	[67]
	Resorbable (Bio-Gide^®^)	Autogenous	Clinical Trial	seven patients with nine implants	2.3 ± 1.6	2.6 ± 1.6	3 yr	2001	Khoury	[25]
	Resorbable (OsseoGuard^®^)	Autogenous	Randomized Clinical Trial	16 patients with 25 implants	−0.7 ± 1.5	1.7 ± 1.8	5 yr	2022	Aghazadeh	[4]
	Resorbable (OsseoGuard^®^)	Autogenous or Bio-Oss^®^	Randomized Clinical Trial	39 patients with 74 implants	1.2 ± 0.5 (95% CIs: 0.1, 2.4)	2.4 ± 0.5 (95% CIs: 1.3, 3.5)	1 yr	2020	Aghazadeh	[39]
	Resorbable (Osgide^®^)	Alloplast (Osbone^®^)	Prospective Case Series	43 patients with 43 implants	2.6 ± 0.1	3.2 ± 1.1	1 yr	2021	González Regueiro	[55]
	Resorbable (Creoss^®^)	Autogenous + Bio-Oss^®^	Prospective Case Series	15 patients with 27 implants	2.2 ± 0.4	3.9 ± 0.2	1 yr	2020b	Monje	[47]
	Resorbable (Bio-Gide^®^)	Bio-Oss^®^	MultiCenter Randomized Clinical Trial	34 patients with 37 implants	2.7 ± 1.3	1.9 ± 1.5	1 yr	2021	Renvert	[34]
	Resorbable (Bio-Gide^®^)	Bio-Oss^®^	MultiCenter Randomized Clinical Trial	30 patients with 59 implants	2.1 ± 1.3	1.6 ±1.9	3 yr	2024	Renvert	[3]
	Resorbable (Bio-Gide^®^)	Bio-Oss^®^	Prospective Case Series	11 patients with 11 implants	2.8 ± 1.5	4.1 ± 0.5	1 yr	2014	Matarasso	[31]
	Resorbable (Bio-Gide^®^)	Bio-Oss^®^	Randomized Clinical Trial	20 patients with 20 implants	2.4 ± 1.4	3.7 ± 1.9	1 yr	2023	Heitz-Mayfield	[38]
	Resorbable (Bio-Gide^®^)	Bio-Oss^®^	Randomized Clinical Trial	21 patients with 21 implants	1.5 ± 2.2	4.5 ± 2.6	1 yr	2023	Regidor	[37]
	Resorbable (Bio-Gide^®^)	Bio-Oss^®^	Randomized Clinical Trial	26 patients with 26 implants	2.0 ± 0.8	2.7 ± 0.4	1 yr	2018	Isler	[32]
	Resorbable (CGF)	Bio-Oss^®^	Randomized Clinical Trial	26 patients with 26 implants	1.6 ± 1.0	2.2 ± 0.2	1 yr	2018	Isler	[32]
	Resorbable (OsseoGuard^®^)	Bio-Oss^®^	Randomized Clinical Trial	23 patients with 38 implants	1.6 ± 1.8	2.8 ± 1.7	5 yr	2022	Aghazadeh	[4]
	Resorbable (Bio-Gide^®^)	Allograft (Puros^®^)	Prospective Case Series	34 patients with 34 implants	0.5 ± 0.4	1.3 ± 0.4	5 yr	2018	La Monaca	[33]
	Resorbable (RTM)	Allograft	Randomized Clinical Trial	17 patients with 24 implants	1.7 ± 0.7	3.4 ± 1.2	1 yr	2023a and b	Monje	[44,61]
	Resorbable (Ossix Plus^®^)	Allograft + vancomycin and tobramycin	Case Series	13 patients with 17 implants	3.8 ± 0.7	4.2 ± 1.7	1 yr	2018	Nart	[48]
	Resorbable (Osseoquest^®^)	Alloplast (Algipore^®^)	Case Series	12 patients with 16 implants	2.3 ± 1.2	4.2 ± 1.5	1 yr	2007a	Roos-Jansåker	[46]
	Resorbable (Osseoquest^®^)	Alloplast (Algipore^®^)	Prospective Cohort Study	17 patients with 29 implants	1.5 ± 1.2	2.9 ± 2.0	1 yr	2007b	Roos-Jansåker	[63]
	Resorbable (Osseoquest^®^)	Alloplast (Algipore^®^)	Prospective Case–Control Study	17 patients with 29 implants	1.6 ± 1.2	Not Available	3 yr	2011	Roos-Jansåker	[62]
	Resorbable (Osseoquest^®^)	Alloplast (Algipore^®^)	Clinical Trial	13 patients with 23 implants	1.3 ± 1.4	3.0 ± 2.4	5 yr	2014	Roos-Jansåker	[64]
	Resorbable (Cytoplast^®^)	Alloplast (β-TCP + HA)	Randomized Clinical Trial	30 patients with 30 implants	2.1 ± 3.0	2.8 ± 0.9	1 yr	2019	De Tapia	[49]
	Resorbable (Bio-Gide^®^)	Alloplast (HA) + Biologics (EMD^®^)	Case Series	11 patients with 20 implants	1.3 ± 0.6	2.2 ± 0.7	1 yr	2022	Pilenza	[36]
	Resorbable (Bio-Gide^®^)	Bio-Oss^®^ ± Allograft + Biologics (Gem21)	Case Series	38 patients with 51 implants	3.8 ± 1.5	5.4 ± 1.5	3–7.5 yr	2012	Froum	[30]
	Resorbable (Bio-Gide^®^)	BioOss ± Allograft + Biologics (Gem21S^®^)	Case Series	100 patients with 168 implants	1.8 ± 2.0	5.1 ± 2.2	2–10 yr	2015	Froum	[53]
	Resorbable (Bio-Gide^®^)	BioOss ± Allograft + Biologics (Gem21)	Retrospective Case Series	38 patients with 46 implants	3.6 ± 2.4	6.7 ± 1.6	3–15 yrs	2022	Froum	[35]
	Resorbable	Autogenous + Bio-Oss^®^	Prospective Case Series	29 patients with 24 implants	2.3 ± 1.9 (8 mo)	1.5 ± 1.2 (1 yr)	8 mo–1 yr	2022b	Wen	[68]
**Non-resorbable membrane + Bone Graft ± Biologics**	Non-Resorbable (Gore-Tex^®^)	Autogenous	Clinical Trial	11 patients with 20 implants	2.0 ± 1.9	Not Available	35 mo	2000	Haas	[58]
	Non-Resorbable (e-PTFE)	Autogenous	Clinical Trial	11 patients with 20 implants	3.4 ± 2.4	5.4 ± 3.0	3 yr	2001	Khoury	[25]
	Non-Resorbable (Gore-Tex^®^)	Allograft	Case Reports	One patient with one implant	Not Available	8.0 (SD not available)	1 yr	1995	Mellonig	[57]
				One patient with one implant	6.0 (SD not available)	8.0 (SD not available)	8 mo			
				One patient with one implant	Not Available	6.0 ± 1.0	1 yr			
	Non-Resorbable (Gore-Tex^®^)	Autogenous + Allograft + Xenograft	Prospective Controlled Study	22 patients with 30 implants	3.5 ± 0.4 (8 mo)	2.9 ± 0.3 ( 1 yr)	8 mo–1 yr	2022a	Wen	[69]
**Non-Resorbable Membrane Only**	Non-Resorbable (Polypropylene)	-	Case Report	One patient with two implants	Not Available	Not Available	4 yr	2022	Bacaro	[59]
	Non-Resorbable (e-PTFE)	-	Case Series	Two patients with two implants	2.3 (SD not available)	3.2 (SD not available)	1 yr	1995	Hämmerle	[27]

Abbreviations: EMD, enamel matrix derivative; CGF, concentrated growth factor; HA, hydroxyapatite; β-TCP, beta-tricalcium phosphate; e-PTFE, expanded polytetrafluoroethylene; GEM21, growth factor enhanced matrix; RTM, resorbable type membrane; mo, month(s); yr, year(s); SD, standard deviation; PPD, probing pocket depth; CIs, confidence intervals. Bio-Oss^®^ & Bio-Oss Collagen^®^ (Geistlich Pharma AG, Wolhusen, Switzerland); Puros^®^ (Zimmer Biomet, Warsaw, IN, USA); Ossix Plus^®^ (Datum Dental, Yavne, Israel); Cytoplast^®^ (Osteogenics Biomedical, Lubbock, TX, USA); Creoss^®^ (Nobel Biocare, Yorba Linda, CA, USA); OsseoQuest^®^ and Gore-Tex^®^ membranes (W. L. Gore & Associates, Newark, DE, USA); Endobon^®^ (Zimmer Biomet, Berlin, Germany); Algipore^®^ (Dentsply Sirona, Bensheim, Germany); GEM 21S^®^ (Lynch Biologics, Franklin, TN, USA); Emdogain^®^ (Straumann Group, Basel, Switzerland); Osbone^®^ (curasan AG, Kleinostheim, Germany); Osgide^®^ (curasan AG, Kleinostheim, Germany).

**Table 2:** Comparison of Average Bone-Fill gain and PPD reduction among Different Treatment Approaches.

	Treatment Modality	Sample Size	Weighted Mean	SD	Standard Error	95% CIs
**Average Bone-Fill Gain (mm)**	^a^ Membrane	34	2.4	1.2	0.2	(2.0, 2.8)
	^b^ No Membrane	12	2.6	1.8	0.5	(1.6, 3.6)
	^c^ Resorbable Membrane	27	2.1	1.0	0.2	(1.7, 2.4)
	^d^ Non-Resorbable Membrane	5	3.4	1.6	0.7	(2.1, 4.8)
	^e^ Resorbable Membrane + Bone Graft ± Biologics	27	2.1	1.0	0.2	(1.7, 2.4)
	^f^ Bone Graft and Biologics Only	1	4.3	0.5	0.5	(3.3, 5.3)
	^g^ Resorbable Membrane + Bone Graft	22	2.1	0.9	0.2	(1.7, 2.5)
	^h^ Resorbable Membrane + Bone Graft + Biologics	4	2.6	1.3	0.6	(1.4, 3.9)
**Average PPD Reduction (mm)**	^a^ Membrane	33	3.5	1.8	0.3	(2.9, 4.1)
	^b^ No Membrane	12	3.7	1.1	0.3	(3.0, 4.3)
	^c^ Resorbable Membrane	26	3.2	1.3	0.3	(2.7, 3.7)
	^d^ Non-Resorbable Membrane	6	5.6	2.2	0.9	(3.8, 7.4)
	^e^ Resorbable Membrane + Bone Graft ± Biologics	26	3.2	1.3	0.3	(2.7, 3.7)
	^f^ Bone Graft and Biologics Only	1	5.4	1.4	1.4	(2.7, 8.1)
	^g^ Resorbable Membrane + Bone Graft	22	3.0	1.0	0.2	(2.5, 3.4)
	^h^ Resorbable Membrane + Bone Graft + Biologics	4	4.9	1.9	1.0	(3.0, 6.7)

^a^ studies using any membrane type; ^b^ studies without membranes; ^c^ studies using resorbable membranes; ^d^ studies using non-resorbable membranes; ^e^ studies combining resorbable membranes and bone grafts with or without biologics; ^f^ studies using bone grafts with or without biologics; ^g^ studies combining resorbable membranes and bone grafts; ^h^ studies combining resorbable membranes, bone grafts, and biologics. Abbreviations: PPD, probing pocket depth; SD, standard deviation; CIs, confidence intervals.

**Table 3:** Comparison of KM Effects in Membrane-Assisted Regenerative Therapy for PI management.

KM	Membrane Type	Bone Graft Type	Study Model	Sample Size	Average Bone-Fill Gain ± SD (mm)	PPD Reduction ± SD (mm)	Period	Year	Author	Reference
**≥2 mm**	Resorbable (Bio-Gide^®^)	Bio-Oss^®^	Prospective Case Series	20 patients with 28 implants	-	1.6 ± 1	2 yr	2022	Obreja	[73]
	Resorbable (Bio-Gide^®^)	Bio-Oss^®^	Prospective Case Series	20 patients with 28 implants	-	0.86 ± 1	2 yr	2020	Galarraga-Vinueza	[74]
	Resorbable (Creoss^®^)	Autogenous + Bio-Oss^®^	Prospective Case Series	15 patients with 27 implants	2.2 ± 0.4	3.9 ± 0.2	1 yr	2020b	Monje	[47]
	Resorbable (Bio-Gide^®^)	Allograft (Puros^®^) or Bio-Oss^®^	Retrospective Study	40 patients with 29 implants	0.6 (SD not available)	2.6 (SD not available)	1–12 yr	2020	Ravidà	[72]
	Resorbable (RTM)	Allograft	Randomized Clinical Trial	17 patients with 24 implants	1.7 ± 0.7	3.4 ± 1.2	1 yr	2023a	Monje	[61]
	Resorbable (Ossix Plus^®^)	Allograft + antibiotics	Case Series	13 patients with 17 implants	3.8 ± 0.7	4.2 ± 1.7	1 yr	2018	Nart	[48]
**<2 mm**	Resorbable (Bio-Gide^®^)	Allograft (Puros^®^) or Bio-Oss^®^	Retrospective Study	40 patients with 39 implants	0.4 (SD not available)	1.4 (SD not available)	1–12 yr	2020	Ravidà	[72]

Abbreviations: KM, keratinized mucosa; PI, Peri-Implantitis; yr, year(s); SD, standard deviation.

**Table 4:** Clinical Outcomes of Implantoplasty in Membrane-Assisted Regenerative Therapy.

Implantoplasty/Surface Protocol	Membrane Type	Bone Graft Type	Sample Size (Number of Implants)	Period (Months)	Average Bone-Fill Gain (mm)	PPD Reduction (mm)	Key Findings	Year	Author	Reference
Implantoplasty (Diamond + Arkansas burs)	Resorbable (Cytoplast)	Alloplast (β-TCP + HA)	30 (15 test/15 control)	12	2.5 ± 1.2 (test)/0.7 ± 1.3 (control)	4.87 ± 1.55 (test)/2.85 ± 1.91 (control)	Ti-brush adjunct to implantoplasty significantly enhanced bone-fill and PPD reduction. Both groups underwent implantoplasty.	2019	De Tapia	[49]
Implantoplasty (Rotary burs)	Resorbable (Bio-Gide^®^)	Bio-Oss^®^	28	12	-	0.86 ± 1	Laser-assisted implantoplasty improved soft-tissue integration and marginal bone stability.	2020	Galarraga-Vinueza	[74]
Implantoplasty (Fine diamond burs)	Resorbable (Osgide^®^)	Alloplast (Osbone^®^)	43	12	2.6 ± 0.1	3.2 ± 1.1	Polished implantoplasty surface yielded greater bone-fill and soft-tissue integration.	2021	González Regueiro	[55]
Implantoplasty (Diamond burs + Arkansas + silicone polishers)	Resorbable (Bio-Gide^®^)	Bio-Oss^®^	11	12	2.8 ± 1.5	4.1 ± 0.5	Combined resective + regenerative approach showed ~93% defect fill and marked PPD reduction.	2014	Matarasso	[31]
Implantoplasty + soft-tissue conditioning	-	No graft	31	12	-	3.0 ± 0.7	Soft-tissue conditioning with implantoplasty achieved 87% disease resolution when KM ≥ 2 mm.	2020a	Monje	[71]
Implantoplasty (Meisinger burs)	Resorbable (Creoss^®^)	Autogenous + Bio-Oss^®^	27	12	2.2 ± 0.4	3.9 ± 0.2	Submerged healing with MB and implantoplasty achieved ~85% disease resolution.	2020b	Monje	[47]
Implantoplasty (Tungsten carbide bur (for uncontained defects))	Resorbable (RTM)	Allograft	24	12	1.7 ± 0.7	3.4 ± 1.2	Defect angle < 40° predicted greater bone gain; implantoplasty improved surface stability.	2023b	Monje	[44]
Implantoplasty + Er:YAG	Resorbable	Bio-Oss^®^	21	48	-	1.2 ± 1.9	4-year follow-up: implantoplasty + bone graft maintained stable results; no difference between laser and manual decontamination.	2013	Schwarz	[77]
Implantoplasty + Er:YAG	Resorbable	Bio-Oss^®^	15	84	-	0.74 ± 1.89	7-year data: PPD and CAL gains stable; surface decontamination method not determinant.	2017	Schwarz	[78]
Implantoplasty + Ti-brush	Resorbable	Bio-Oss^®^ (collagen/spongiosa)	20	12	-	1.2 ± 0.5	Ti-brush improved surface cleanliness and healing in the combined regenerative approach.	2023	Schwarz	[79]
Implantoplasty at the supracrestal site	Resorbable	Allograft	24 patients (12 test/12 control)	6	1.27 ± 1.14 (test)/1.08 ± 1.04 (control)	2.65 ± 2.14 (test)/1.85 ± 1.71 (control)	The laser-assisted group showed greater PPD reduction.	2021	Wang	[56]
Implantoplasty + Er:YAG laser	Resorbable	Allograft	24	30	2.82 ± 0.46 (Laser)/1.96 ± 0.46 (control)	3.04 ± 1.0 (Laser)/1.84 ± 1.0 (control)	The laser group maintained greater PPD reduction and bone gain.	2023	Wang	[52]
Implantoplasty (rotary + air-abrasive)	Non-Resorbable (Gore-Tex^®^)	Autogenous + Allograft + Xenograft	30	8–12	3.5 ± 0.4 (8 months)	2.9 ± 0.3 ( 1 year)	Submerged regenerative protocol achieved ≈ 3 mm bone gain and ≈ 3 mm PPD reduction.	2022a	Wen	[69]
Implantoplasty (Rotary + air-abrasive)	Resorbable	Autogenous + Bio-Oss^®^	24	8–12	2.3 ± 1.9 (8 months)	1.5 ± 1.2 (1 year)	Non-submerged regenerative protocol (with crown removal) achieved significant bone gain and PPD reduction.	2022b	Wen	[68]
Implantoplasty (rotary Meisinger system)	Resorbable	Autogenous + Allograft + Xenograft	59 implants (30 submerged/29 non-submerged)	12	Submerged: 3.22 ± 0.41/non-submerged: 2.33 ± 1.88	Submerged: 2.93 ± 0.25/non-submerged: 1.51 ± 1.17	The submerged approach showed 1.3 mm PPD gain compared to the non-submerged.	2024	Wen	[80]
Implantoplasty (diamond under prosthesis)	-	Autogenous + demineralized xenograft	36	12	3.5 (SD not available)	4.0 (SD not available)	Etching-gel decontamination + autogenous/xenograft mix provided 3.5 mm bone gain and 4 mm PPD reduction at 1 year.	2012	Wiltfang	[65]

Abbreviations: Er:YAG, Erbium-doped Yttrium Aluminum Garnet Laser; Ti-brush, Titanium Brush; β-TCP, Beta-Tricalcium Phosphate; RTM, Resorbable Type Membrane; SD, standard deviation; PPD, probing pocket depth.

## Data Availability

The original contributions presented in this study are included in the article/Supplementary Materials. Further inquiries can be directed at the corresponding authors.

## Supplementary Materials

The following supporting information can be downloaded at: https://www.mdpi.com/article/10.3390/ma18225227/s1, Table S1. PRISMA 2020 checklist for reporting systematic reviews and meta-analyses. Table S2. Summary of studies reporting membrane-assisted regenerative therapy and bone graft-based approaches in PI management. The table presents follow-up duration, study design, membrane and graft types, bone-fill gain, PPD reduction, KM width, prosthesis removal or implantoplasty status, and decontamination protocols. Abbreviations: PPD, probing pocket depth; KM, keratinized mucosa; GTR, guided tissue regeneration; RTM, resorbable type membrane; e-PTFE, expanded polytetrafluoroethylene; dPTFE, dense polytetrafluoroethylene; DBBM, deproteinized bovine bone mineral; FDBA, freeze-dried bone allograft; β-TCP, beta-tricalcium phosphate; HA, hydroxyapatite; EMD, enamel matrix derivative; CGF, concentrated growth factor; Er:YAG, erbium-doped yttrium aluminum garnet laser Table S3. Weighted comparison of average bone-fill gain in preclinical and clinical studies evaluating non-resorbable membranes in membrane-assisted regenerative therapy for PI management. Abbreviations: HA, hydroxyapatite; mo, month(s); yr, year(s); SD, standard deviation; CIs, confidence intervals Figure S1. Risk-of-bias assessment for randomized controlled trials using the Cochrane RoB tool. Abbreviation: RoB, Risk of Bias Figure S2. Forest plots comparing membrane-assisted and non-membrane-assisted regenerative approaches for peri-implantitis. (A) Mean differences in average bone-fill gain. (B) Mean differences in PPD reduction. Each square represents an individual study, while the diamond indicates the pooled mean difference with a 95% CIs. Both analyses demonstrated statistically significant improvements favoring membrane-assisted regenerative therapy (*p* < 0.001); however, high heterogeneity (I^2^ > 80%) was observed across studies. Abbreviation: PPD, probing pocket depth Figure S3. Funnel plots assessing potential publication bias among the included studies. (A) Funnel plot for bone-fill gain outcomes. (B) Funnel plot for PPD reduction outcomes. Both plots exhibit near-symmetrical distributions around the mean effect size, indicating minimal publication bias and supporting the overall robustness of the pooled estimates. Abbreviation: PPD, probing pocket depth.

## Abbreviations

PPD, probing pocket depth; GBR, guided bone regeneration; PRISMA, Preferred Reporting Items for Systematic Reviews and Meta-Analyses; RoB, Risk of Bias; PI, Peri-implantitis; BOP, bleeding on probing; e-PTFE, expanded polytetrafluoroethylene; KM, keratinized mucosa; GM: Gingival Margin; aJE: Apical extent of Junctional Epithelium; aICT: Apical extent of Inflammatory Cell Tissue; BC: Bone Crest; DBBM, deproteinized bovine bone mineral; PRF, platelet-rich fibrin; EMD, enamel matrix derivative; CGF, concentrated growth factor; HA, hydroxyapatite; β-TCP, beta-tricalcium phosphate; GEM21, growth factor enhanced matrix; RTM, resorbable type membrane; SD, standard deviation; PDL, Periodontal ligament; CT, Connective tissue; 3D, 3-dimensional; FDBA, freeze-dried bone allograft; dPTFE, dense polytetrafluoroethylene; mo, month(s); yr, year(s); CIs, confidence intervals; PCL, polycaprolactone; H_2_O_2_, Hydrogen Peroxide; Ti-brush, Titanium Brush; CHX, Chlorhexidine; Er:YAG, Erbium-doped Yttrium Aluminum Garnet Laser.
