# Supplementary material for: Evaluating the Clinical Efficacy of Membrane-Assisted Regenerative Therapy in Peri-Implantitis Management: A Comprehensive Review Incorporating Systematic Review Evidence
Source: Materials (Basel). 2025 Nov 18;18(22):5227. doi: 10.3390/ma18225227 (PMC12654031; doi:10.3390/ma18225227)
Supplement: Supplementary file 1 [file materials-18-05227-s001.zip › Table S3.pdf]

Table S3: Weighted comparison of average bone-fill gain in preclinical and clinical studies evaluating non-resorbable membranes in membrane-assisted regenerative therapy for PI management.

Preclinical Study								
**Membrane Type**	**Bone Graft Type**	**Average Bone-Fill Gain(mm)**	**Sample Size**	**Defect Type**	**Period**	**Year**	**Author**	**Reference**
Non-resorbable (Gore-tex®)	Resorbable HA	2.6 ± 0.4	7	-	5mo	1997	Hürzeler	[96]
Non-resorbable (Gore-tex®)	Autogenous	4.7 (SD not available)	16	-	6mo	2003a	Schou	[93]
Non-resorbable (Gore-tex®)	Autogenous	4.4–4.7 (SD not available)	64	-	6mo	2003b	Schou	[97]
Non-resorbable (Gore-tex®)	Bio-Oss®	5 (SD not available)	16	-	6mo	2003c	Schou	[94]
**Weighted Mean**	4.511	**Pooled SD**	0.4	**SE**	0.039	**95% CIs**	(4.433, 4.588)	

Clinical Study								
**Membrane Type**	**Bone Graft Type**	**Average Bone-Fill Gain(mm)**	**Sample Size**	**Defect Type**	**Period**	**Year**	**Author**	**Reference**
Non-resorbable (Gore-tex®)	Autogenous	2.0 ± 1.9	24	-	35mo	2000	Haas	[58]
Non-resorbable (Gore-tex®)	Autogenous	3.4 ± 2.4	20	-	3yr	2001	Khoury	[25]
Non-resorbable (Gore-tex®)	Allograft	6 (SD not available)	1	wide 3-walled osseous defect	8mo / 1yr	1995	Mellonig	[57]
Non-resorbable (Gore-tex®)	Autogenous + Allograft + Xenograft	3.5 ± 0.4	30	surrounding bony walls	8mo–1yr	2022a	Wen	[69]
**Weighted Mean**	3.027	**Pooled SD**	1.666	**SE**	0.192	**95% CIs**	(2.65, 3.404)	
