# Supplementary figures and images for: Evaluating the Clinical Efficacy of Membrane-Assisted Regenerative Therapy in Peri-Implantitis Management: A Comprehensive Review Incorporating Systematic Review Evidence
Source: Materials (Basel). 2025 Nov 18;18(22):5227. doi: 10.3390/ma18225227 (PMC12654031; doi:10.3390/ma18225227)

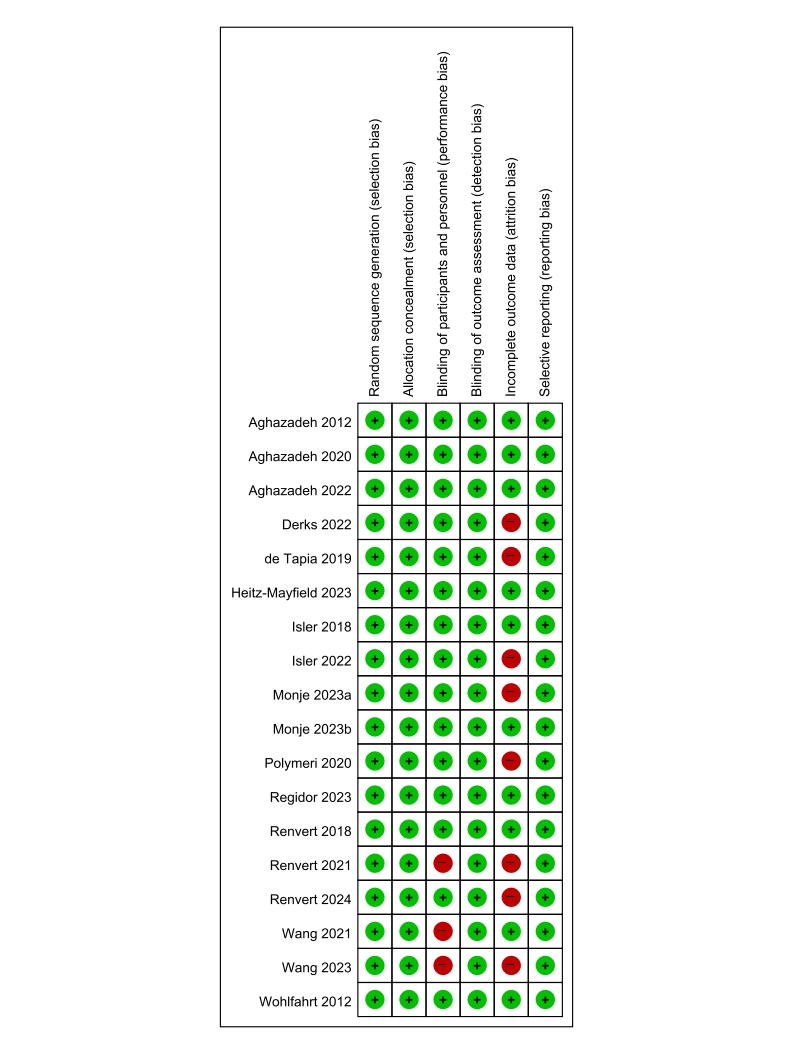

Supplement: Supplementary file 1 [file materials-18-05227-s001.zip › Figure S1.jpg]

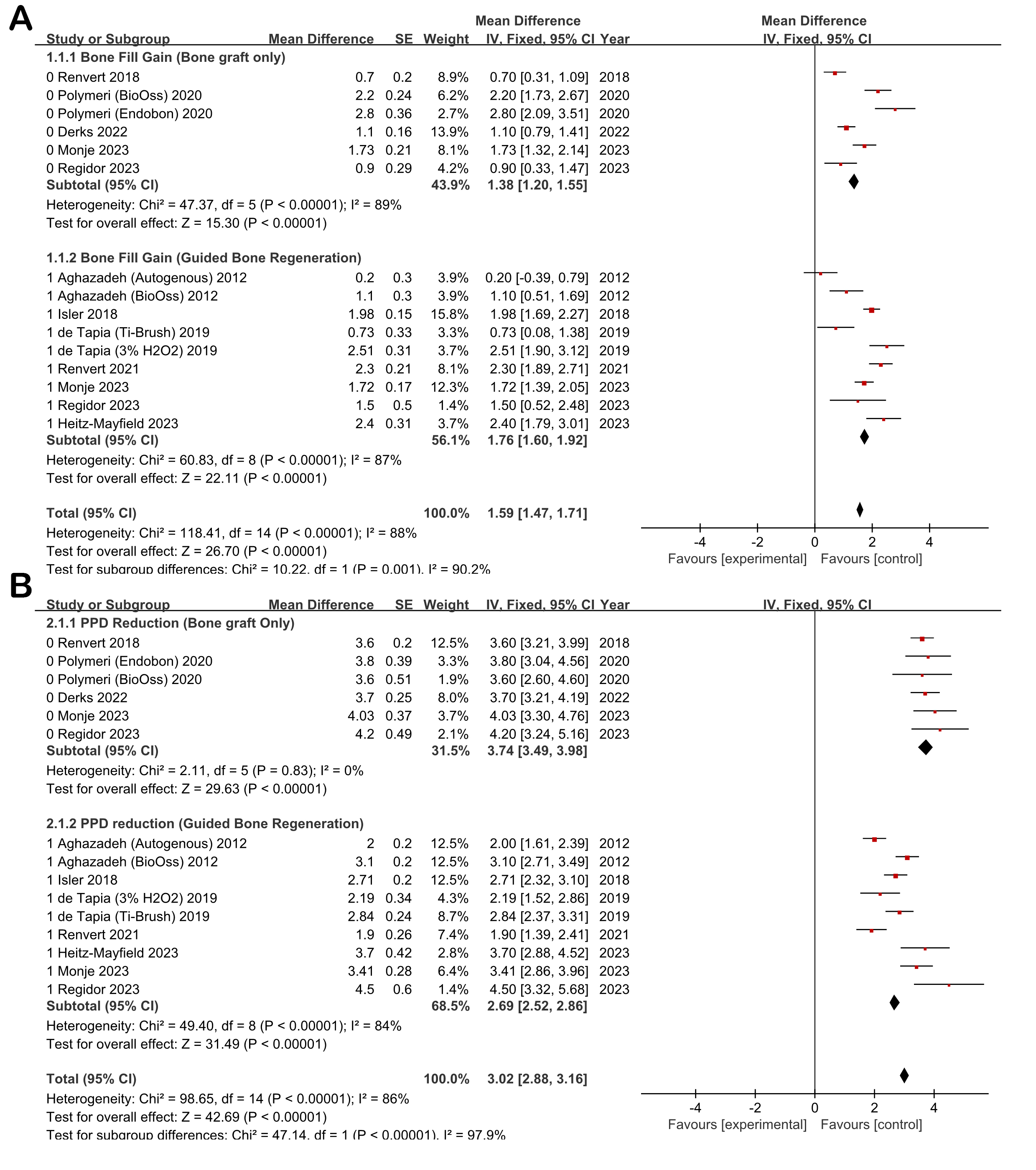

Supplement: Supplementary file 1 [file materials-18-05227-s001.zip › Figure S2.jpg]

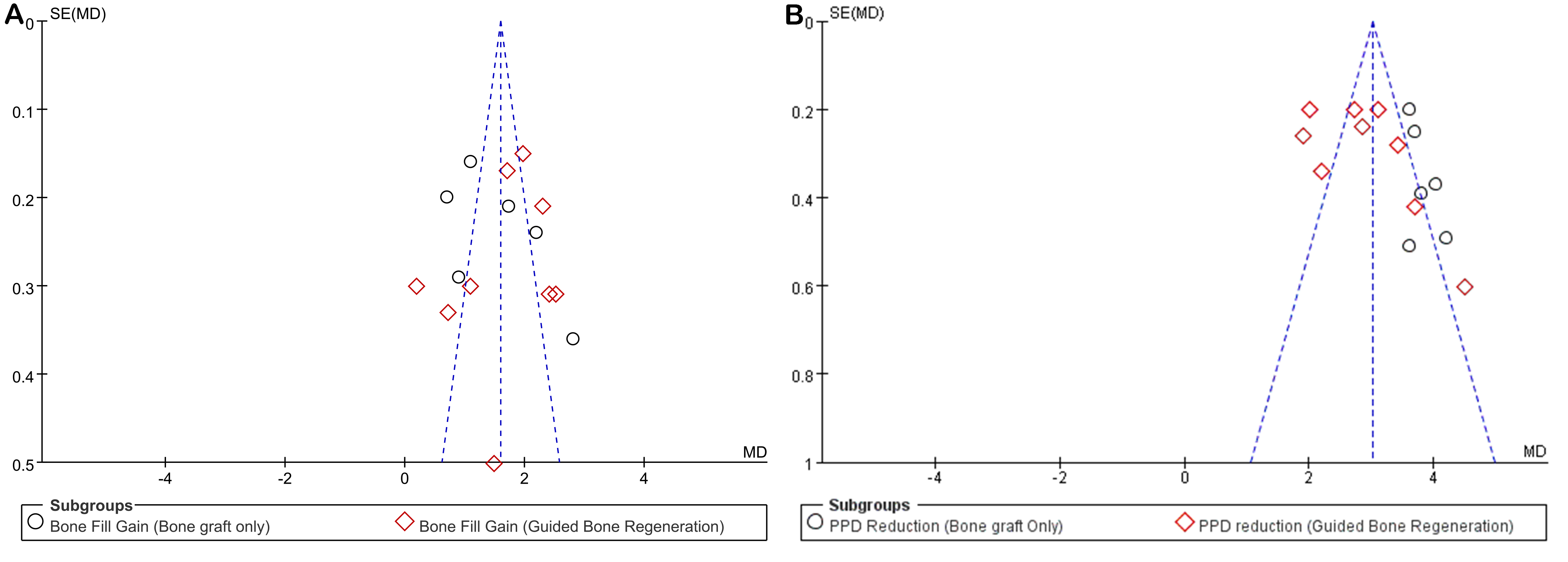

Supplement: Supplementary file 1 [file materials-18-05227-s001.zip › Figure S3.jpg]
